# Comparisons of historical Dutch commons inform about the long-term dynamics of social-ecological systems

**DOI:** 10.1371/journal.pone.0256803

**Published:** 2021-08-27

**Authors:** Anders Forsman, Tine De Moor, René van Weeren, Mike Farjam, Molood Ale Ebrahim Dehkordi, Amineh Ghorbani, Giangiacomo Bravo

**Affiliations:** 1 Department of Biology and Environmental Science, Linnaeus University, Kalmar, Sweden; 2 Rotterdam School of Management, Erasmus University, Rotterdam, The Netherlands; 3 Department of History and Art History, Utrecht University, Utrecht, The Netherlands; 4 European Studies, Centre for Languages and Literature, Lund University, Lund, Sweden; 5 Faculty of Technology, Policy and Management, Delft University of Technology, Delft, The Netherlands; 6 Department of Social Studies and Centre for Data Intensive Sciences and Applications, Linnaeus University, Växjö, Sweden; Utah State University, UNITED STATES

## Abstract

Human societies and natural ecosystems are under threat by growing populations, overexploitation of natural resources and climate change. This calls for more sustainable utilization of resources based on past experiences and insights from many different disciplines. Interdisciplinary approaches to studies of historical commons have potential to identify drivers of change and keys to success in the past, and offer advice about the management and use of shared resources in contemporary and future systems. We address these issues by applying an ecological perspective to historical data on social-ecological systems. We perform comparisons and time series analyses for nine successful Dutch commons for which high-resolution data on the regulatory activities and use of shared resources is available for on average 380 years (range 236 to 568) during the period 1300 to 1972. Within commons, institutional developments were oscillating, with periods of intense regulatory activity being separated by periods of low activity, and with the dynamics of regulations being largely independent across commons. Ecological theory posits that species that occupy similar niches should show correlated responses to environmental challenges; however, commons using more similar resources did not have more parallel or similar institutional developments. One notable exception was that sanctioning was more frequent in commons that directed more regulatory activities towards non-renewable subsoil resources, whereas there was no association between sanctioning and the use of renewable resources. This might indicate that commoners were aware of potential resource depletion and attempted to influence freeriding by actively trying to solve the underlying social dilemmas. Sanctioning regulations were more frequent during the first than during the second part of a common’s life, indicating that while sanctioning might have been important for the establishment of commons it was not key to the long-term persistence of historical commons.

## Introduction

A growing human population, exploitation, changes in land use, habitat fragmentation and degradation together with ongoing climate change impose severe threats to biodiversity, ecosystems and the human societies that depend on them. Throughout history, the set-up of institutions for collective action, created for the management and use of resources among the members of the collectivity [1,2] has been directed towards collectively beneficial outcomes whilst overcoming conflicts of interest and potential selfish short-term temptations associated with social dilemmas, also referred to as the Tragedy of the Commons [3–8]. Despite a growing scientific interest in the management of natural resources under collective governance regime [9–14], important aspects of the dynamics and drivers of long-term development of commons-institutions, often spanning several centuries, remain largely unexplored.

In the study reported here, we analyze and compare patterns of long-term institutional change and resource use in self-governed medieval and early modern Dutch commons that were established for the utilization and management of shared resources (e.g., infrastructure, vegetation, animals, and subsoil resources such as peat). The development of commons is likely reflective of intrinsic factors, such as the nature of the shared resources, and the number and background of the commoners, external environmental conditions such as natural disasters, weather conditions and climate change, and external social, political and economic pressures [15]. However, attempts to quantitatively study patterns and to identify drivers of institutional dynamics of commons remain scarce [9,15–17]. Although commons have over the past 20 years increasingly been considered as socio-ecological systems [18], the parallels that can be drawn on a more abstract level between eco-evolutionary developments of biological populations, species, and ecosystems and those of commons as institutions are largely absent from literature [19]. Yet, theories and approaches developed in ecological and evolutionary research have potential to further understanding, vitalize research, and to inform about the patterns and drivers of spatiotemporal variation in the development of historical commons, with regards to rules, regulations and decision-making [15,16,20,21]. For example, previous analyses of more than 400 Dutch commons over more than a millennium uncovered that most commons in that region originated between 1200 and 1700, and that there was a particularly high rate of evolution during 1300–1550, a pattern intermediate to gradualism and punctuated equilibrium in biological evolution [15]. Similarly, dissolutions of commons were rare prior to 1800 and peaked around 1850, comparable to a mass extinction in biology, whereas temporal trends in number and spatial distribution of commons resembled patterns of growth of biological communities and populations, showing signs of saturation determined by the abundance and distribution of resources [15].

Ecological and evolutionary theory posit that species using similar resources and environments (i.e., occupy similar niches) should face similar challenges and selective regimes, and therefore converge on a shared evolutionary solution [22,23] and show correlated population dynamic responses to environmental changes [24,25]. Conversely, species that occupy different niches are expected to respond independently because their past histories and preconditions are unique, and their key drivers largely unrelated. Farjam, de Moor [9] recently analyzed institutional regulatory activities in commons in the Netherlands and the UK, and they report a U-shaped pattern with the strongest activity in the first quarter and towards the end of a commons’ life span, with a period of reduced activity in between. However, a more in-depth analysis is required to determine whether the identified pattern of institutional development is generalizable across commons or whether responses are uncorrelated and vary according to the time period or composition of shared resources utilized by the commoners.

In biological systems, populations and species that are exposed to changing environmental conditions and altered selection regimes may respond by micro-evolutionary modifications in the form of adaptations brought about by shifts between generations in the frequencies of alleles (gene variants) that influence the phenotypic properties of individuals [26–28]. When conditions are stable, however, the phenotypic values and genetic architecture of populations should remain largely unchanged, save for the stochastic effects of genetic drift [29,30]. Besides evolutionary shifts, individuals and populations may respond to environmental changes via intra-individual, reversible, phenotypic flexibility and developmental plasticity, whereby the phenotypic expression of genetic variants is modulated by the environmental conditions that individuals experience during development and growth (i.e., phenotypic changes that do not require genetic modifications) [31–33]. To our knowledge, it has not been investigated whether patterns of long-term institutional change in historical commons best resemble evolutionary adaptations (manifest as additions and modifications of written rules and regulations) or whether commons managed to cope with challenges via a strategy resembling more closely phenotypic flexibility and developmental plasticity (i.e., without adding or changing the rules and regulations that form the institutions of commons). Understanding such mechanisms can help regulators identify commons under pressure and develop coherent policies for supporting commons.

Here, we analyze and compare the development of historical commons institutions. To that end, we use data for nine Dutch commons spanning over 650 years (from 1300 to 1972) collected within the framework of the “Common Rule-Project” (CRP) [11]. These nine cases are the only ones for which the information put together during the inventory of archival material in the early 20th century and reported in the atlas of commons [11] meet all the requirements of our analyses; information on start and end date, variation in longevity, information on the types of resources used, and, most importantly, continuous detailed records of the decisions (rules) made by the commoners to manage the resources for the whole time-span of the commons. The regulatory activities regarding rules as formulated and discussed by the commoners were all about resources that the commoners managed and used collectively, as a group of commoners, not as a society (public goods) or as an individual (private goods). Specifically, this extended dataset captures the institutional rules which commoners regularly established, updated, or changed to foster cooperation and to manage the use of shared resources. A limitation of this approach is that not all modifications and changes are captured in the formal records, independently of how detailed they are. For instance, Ostrom (1) points out to the importance of informal institutions and various types of rules-in-use for the sustainable management of commons. Unfortunately, the availability of this kind of data in historical record is severely limited, especially for older times. At the same time, the formal records available for the nine Dutch cases included in the present study are relatively rich and can still provide a rather nuanced picture of the management activities.

Using these data we first explore whether institutional developments in different commons are independent or correlated. Next, we evaluate the hypothesis that temporal distributions of regulatory activities (rule changes) are more similar and synchronized between commons that utilize similar resources, compared with commons that rely on dissimilar resources. Thereafter, we evaluate the hypothesis that enforcement mechanisms such as sanctioning have been disproportionally used to manage harvesting of non-renewable resources. Lastly, we evaluate whether the frequent use of sanctioning related regulations is associated with increased or decreased lifespan of the commons.

## Methods

### Dutch commons and sources of data

The analyses outlined below were conducted based on data for nine Dutch commons, referred to as *Marken*. These Marken were a type of purely self-governed commons, with little or no interference from other parties than the commoners themselves in the use and management of resources (contrary to for example Meenten, elsewhere in the Netherlands, where the local government interfered substantially). The Marken were established in particular in the eastern and northern part of the Netherlands. These independent commons were essentially associations of farmers, which had boards or steering committees especially created for the purpose of the management of the collective resources, and with decision making taking place at regular (yearly, or more frequent) meetings, sometimes with compulsory attendance. During these meetings, the commoners developed and amended the institutional rules to facilitate the maintenance and use of the shared resources. Entitlement to use the common was linked to being a legal inhabitant of the area the common belonged to and/or the possession of land or real estate in the area concerned.

The nine historical commons from The Netherlands used for this study each have an extensive and reliable written documentation [11] that was used for the analyses of long-term institutional dynamics. The first archival sources of the majority of the *Marken* in the Netherlands date back to the late Middle Ages or early modern times. Our data for the nine commons cover more than 650 years, from 1300 to 1972. All selected cases had records that lasted for at least two hundred years. Admittedly, the dataset used for this study has an implicit bias, in that it includes only successful commons that survived for over 200 years. Such long life spans were not unusual, however; results from previous analyses based on data for a much larger number of Dutch commons show that commons in the past overall were long-lived (mean ± s.d. = 371 ± 200 years, range 9–1110 years, *n* = 351) [15]. It is nevertheless possible that the dynamics of short-lived commons may have shown patterns different from those reported here.

**Fig 1:**
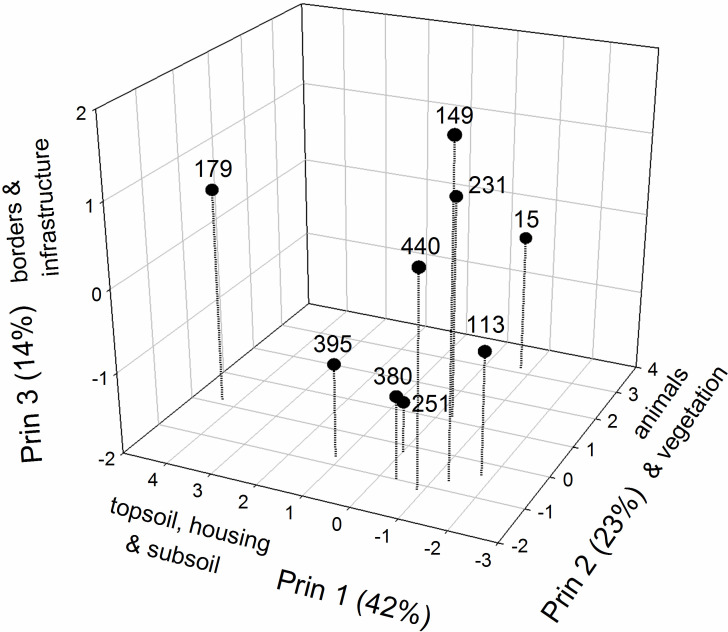
Visual representation of the separation of Dutch commons according to the number of regulatory activities pertaining to different types of shared resources. The first three principal components accounted for 79% of the total variance. According to eigenvectors (S2 Table) large positive values on Prin 1 reflect high importance of topsoil, housing, subsoil and unspecified resources; large positive values on Prin 2 reflect high importance of animals and vegetation resources; and large values on Prin 3 reflect high importance on borders and infrastructure. **Key to Common IDs** (15 = Mark Geesteren, Mander en Vasse, 113 = Mark Berkum, 149 = Mark het Gooi, 179 = Mark Exel, 231 = Dunsborger Hattemer mark, 251 = Mark Coevorden, 380 = Mark Bestmen, 395 = Mark Rozengaarde, 440 = Mark Raalterwold.

Eight out of our nine Dutch commons were located in or just outside the current province of Overijssel. The geographical outlier in our dataset is the Marke Het Gooi, which was located near Hilversum, in the far southeast corner of the current province of North-Holland. Information on more features of the commons, including size, user types, and a map showing their locations is available in Fig 1 and Appendix 2 in De Moor and Tukker [34]. Groups of commoners regularly created new rules or adapted existing rules on the use, governance and management of resources (henceforth, ‘regulatory activities’). Data on the temporal distributions of such regulatory activities was extracted from the Common Rules Project (CRP) database [11]. The database includes over 800 commons that emerged in the same period and in the same region, and contains a transcription of background information taken from the original archival sources regarding the rules of use, governance and management that were designed for the commons. The nine commons used for this study were all long-lived and the only ones for which archival data on decisions (rule changes) made to manage the resources throughout the lifespan of the common has been compiled that allows for detailed analyses (e.g., [9,34]). Following Ostrom’s work [1,35], the original rules were grouped into three categories, representing resource related, bureaucratic or sanctioning rules [9]. This is a simplified version of the categorization used by De Moor, Laborda-Pemán [11] in the fields Rule Category and Rule Form. A more detailed description of the coding system, transcription of the regulatory activities as taken from the original archival sources, and classification of rules used in the present study is provided in Farjam, de Moor [9]. Temporal distributions of bureaucratic, resource related and sanctioning related regulatory activities were correlated (S1 Fig). We therefore pooled data on the number of rule changes recorded across the three different categories of rules for each year and common, and used this combined measure of regulatory activity as the dependent variable in the statistical analyses, unless otherwise stated. Regulatory activities were assigned to one of eight categories of shared goods, depending on the type of resource that was targeted (Table 1 and S1 Table).

**Table 1:** Characterization of Dutch commons according to the percentage of total number of regulatory activities (rules) that pertain to eight types of resources. A higher resolution characterization of each type of collective resource is provided in S1 Table.

Common name Common ID	Mark Geesteren, Mander en Vasse	Mark Berkum	Mark het Gooi	Mark Exel	Dunsborger Hattemer mark	Mark Coevorden	Mark Bestmen	Mark Rozengaarde	Mark Raalterwold
Resource	15	113	149	179	231	251	380	395	440
Animals	23.05	29.09	71.47	19.95	18.70	30.66	41.67	37.88	23.70
Borders	5.39	3.64	0.82	2.43	1.63	1.89	5.77	8.33	0.93
Housing	4.49	0	0	5.12	6.91	10.85	0	0.76	4.13
Infrastructure	1.80	25.45	1.23	5.39	5.69	11.32	1.92	13.26	9.05
Subsoil Resources	8.98	0	3.02	25.07	19.51	0.94	7.05	0.	10.12
Topsoil Resources	8.38	5.45	3.70	9.70	15.85	3.30	14.10	7.95	20.91
Vegetation	8.68	2.27	4.12	0.54	6.10	2.36	5.77	2.27	1.46
Unspecified	39.22	34.09	15.64	31.81	25.61	38.68	23.72	29.55	29.69
Grand Total %/*N*	100%/334	100%/220	100%/729	100%/371	100%/246	100%/212	100%/156	100%/246	100%/751
Start to end year	1498–1847	1300–1830	1404–1972	1616–1852	1553–1858	1519–1860	1458–1847	1421–1859	1445–1843

### Statistical analysis

Statistical analyses were performed with SAS version 9.4. Spearman rank correlation analysis was used to examine whether the distributions of regulatory activities among years were independent or correlated between pairs of commons. Bonferroni corrections were used to assess statistical significance of correlations (S2 and S3 Tables). To explore also more complex (e.g., periodic) relationships between commons in terms of rule changes, the same associations were checked using the maximal information criterion [36].

To characterize and visualize the differences among commons with regards to the compositions of regulatory activities directed to different types of shared goods a principal component analysis (using procedure PRINCOPM in SAS) was applied to a correlation matrix calculated from the number (not percentages or proportions) of regulatory activities (rules) pertaining to different resource categories (Table 1 and S1 Table). Principal components were not standardized to unit variance but have variances equal to their corresponding eigenvalue. With this approach, the correlations between the original types of resources and the principal components are given by factor loadings (eigenvectors, S4 Table).

To statistically evaluate the dynamics of the regulatory activities, polynomial regressions of degree 3, and lower, were fitted to the data using regression models in which count data on number of regulatory activities per year was treated as response variable. Common identity was treated as a fixed class variable, and linear (Y), quadratic (Y^2^) and cubic (Y^3^) effects of calendar year were treated as continuous predictor variables. To formally test whether institutional development varied among commons, the initial model included the interactions between common ID and the linear, quadratic and cubic effects of year. Models were fitted using procedure GENMOD in SAS. Because the count data was overdispersed and had a high incidence of zeros the response variable was modelled with a zero inflated negative binomial (ZINB) distribution. The scaled Pearson Chi-square statistics and associated *p*-values were used as formal tests for overdispersion. Model selection was based on comparisons between goodness-of-fit (Deviance) and Akaike information criterion (AIC), and statistical significance of interactions and main effects were evaluated by comparisons between models with and without the terms of interest using Likelihood ratio tests (LRT) [37]. To assess robustness of results and conclusions the analyses described above were performed first on the entire data set, then restricted to data on the six commons for which longitudinal times series data is available for ≥ 249 years (common IDs 15, 113, 149, 380, 395 and 440, see Fig 3).

**Fig 2:**
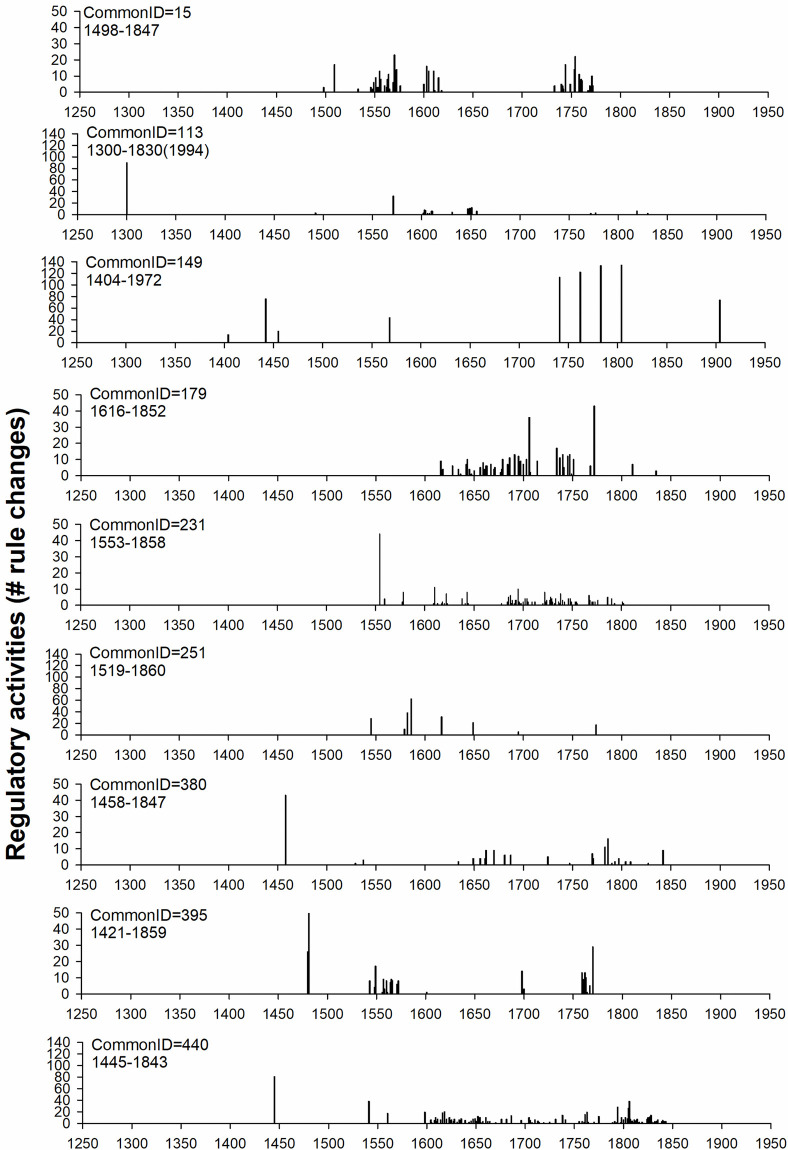
Temporal distributions of regulatory activities (rule changes) for nine Dutch commons. **Key to Common IDs** (15 = Mark Geesteren, Mander en Vasse, 113 = Mark Berkum, 149 = Mark het Gooi, 179 = Mark Exel, 231 = Dunsborger Hattemer mark, 251 = Mark Coevorden, 380 = Mark Bestmen, 395 = Mark Rozengaarde, 440 = Mark Raalterwold.

To quantify differences between pairs of commons in resource management profiles, a distance matrix was computed based on the data in Table 1 using procedure DISTANCE (with settings TYPE = DISTANCE, METHOD = EUCLIDIAN, VARIABLE = INTERVAL and STD = STD) (for the output see S5 Table). Another distance matrix was computed for pairwise differences in temporal distributions of administrative activities (see Fig 2) using procedure DISTANCE (with settings TYPE = DISTANCE, METHOD = EUCLIDIAN, VARIABLE = ORDINAL, and ORDER = DESCENDING) (S6 Table). To examine whether pairs of commons that were built around more similar shared resources had more synchronized institutional dynamics, a matrix correlation analysis (Mantel test) was performed and the 95% CI around the correlation coefficient was estimated using bootstrap (500 permutations). To assess robustness, the matrix correlation was estimated with a Monte-Carlo test in R (with 9999 replications).

To evaluate whether sanctioning related regulations were disproportionately used to manage the use and avoid depletion of non-renewable resources, the associations across commons between the number of sanctioning related regulations per year and the relative importance of non-renewable (peat) versus renewable (vegetation) resources were evaluated using Pearson correlation analysis. Finally, to evaluate whether sanctioning was associated with increased or decreased viability of commons we evaluated the association between the annual rate of sanctioning related regulations and life-span of commons using Pearson correlation analysis.

## Results and discussion

### Characterization of the commons used for analyses of long-term institutional dynamics

The time series of the nine commons for which high resolution data is available span on average 380 years (range 236 to 568). These commons demonstrated a large variety in types of resources available for the use of the commoners: animals, borders, housing, infrastructure (such as roads, but also rivers), subsoil resources (such as peat), topsoil resources (such as grass, or sods), vegetation (varying types of wood resources), and other resources (Table 1). These commons did not specialize on a single resource but resemble instead generalist biological species with relatively broad niches [38]. Previous analyses based on a larger data set indicate that Dutch commons that used more diverse resource types had longer lifespans [15]. To keep members motivated to behave as “good commoners”, it was important that a range of resources was available, in order to ensure that the utility of being a member remained high at all times. The amount of management directed towards different resource types varied within and between commons. For example, the percentage of all regulatory activities pertaining to animals ranged from 20% to 70% (Table 1). This high level of regulatory activity associated with animals should not surprise, as keeping animals on the common was a frequent activity, and usually also the most important form of land use for the commoners. Regulatory activities related to infrastructure ranged from 1 to 25%, and subsoil resources (e.g., peat) ranged from 0% to 70%, per common (Table 1, for high resolution data on number of regulatory activities see S1 Table). A visual representation of the separation of commons according to the amount of management directed towards different resources point to considerable differences in resource use (Fig 1). Below, we explore whether these differences were reflected in the institutional dynamics of the commons.

### Variation in temporal development of institutions within and among commons

The temporal distributions of bureaucratic, resource related, and sanctioning regulatory activities (introduction of new rules, modification and replacement of existing maladapted rules, and the abandonment of superfluous rules) were positively correlated (S1 Fig). The finding that the temporal distributions of different types of regulatory activities (bureaucratic, resource related, and related to sanctioning) went hand in hand might suggest that rule making begets more rule making. To change collective rules, the commoners had to meet. In principle, meetings were held every year, but not all meetings resulted in rules being changed; our data indicate many periods without any activity (Fig 2). When the commoners convened to discuss specific matters it is likely that connected rule changes in various domains might have been deemed necessary. For example, new or adjusted resource related rules may require new or modified sanctioning (e.g. if a resource is overused, it may be effective to increase the fine to reduce freeriding) and additional bureaucracy to make sure the rule is not violated. Results below are based on analyses of the combined activities, unless otherwise stated.

Institutional dynamics in the nine studied Dutch commons from the 14^th^ to the 20^th^ century were not evenly distributed over time (Fig 2). Instead, there were several periods of intensified regulatory activity separated by periods of lower activity (Fig 2). It should be emphasized here that also during the periods of no or low regulatory activity, regular (annual or other) meetings were held by the commoners, but these meetings did not result in any adaptations in form of the addition, modification, replacement or abandonment of rules [15].

Fig 2 shows that the temporal distributions of regulatory activities varied in a complex manner among commons, and that institutional development was typically non-linear and non-generalizable. This was supported by the results from statistical analyses. Model comparisons based on goodness-of-fit statistics, Akaike information criterion (AIC) and Likelihood ratio tests [37] concur that the interaction between common identity and quadratic effects of year, and the interaction between common identity and the cubic effects of year, are significant contributors of variation in regulatory activities that cannot be removed from the model (S7 Table). The overall result and conclusion regarding heterogeneity of institutional change among commons remained largely unchanged when the analyses were restricted to data for the six commons for which longitudinal times series data is available for at least at least 250 years (commons Mark Geesteren Mander en Vasse, Mark Berkum, Mark het Gooi, Mark Bestmen, Mark Rozengaarde, and Mark Ralterwoold, S8 Table).

Regulatory activity was high during the earlier part of the commons life in about half of the cases (Commons Mark Berkum, Dunsborger Hattemer mark, Mark Bestmen and Mark Ralterwold, Fig 2). Not all commons adhered to the pattern of a high initial burst of regulatory activity, with the most notable exceptions being common Mark het Gooi and Mark Exel (Fig 2). Two of the commons (Commons Mark Geesteren Mander en Vasse and Mark Exel) showed a higher activity in the middle than in the beginning or the end of the lifetime of the commons (Fig 2). For common Mark Rozengaarde there was a high activity around 1760 but it was not formally dissolved until one century later, in 1859. Besides the bursts of activity at the beginnings and ends of the life of commons reported previously [9] and above, the data points to four patterns of regulatory activity (Fig 2): *i)* Extended periods (ca 50 years) of intense clumped activity and very low activity in between (Commons Mark Geesteren Mander en Vasse and Mark Rozengaarde); *ii*) Continuous low activity (Commons Dunsborger Hattemer mark and Mark Ralterwold); *iii*) A gradual increase in activity from birth throughout most of the lifespan of the common (Common Mark Exel); and *iv*) Extended periods without regulatory activity interrupted by occasional years with high regulatory activity (Commons Mark het Gooi and Mark Coevorden). Common Mark Exel was the only common with an even temporal distribution of regulatory activities and differed markedly from all the others also in management of resource use (Fig 1), two distinctive features being that management was little directed towards animals while being much concerned with borders and infrastructure compared with the others commons (Table 1). Below, we examine in greater detail whether institutional dynamics were more or less synchronized across commons depending on their resource use, and briefly discuss whether periods of regularly activity and inactivity coincided with the temporal distribution of external and internal challenges.

### Evaluating associations of institutional development among commons

The institutional developments of the nine commons analyzed here were largely independent. Temporal distributions of regulatory activities were significantly and positively correlated in only 4 of the 36 pairwise comparisons (between commons Mark Geesteren Mander en Vasse and Mark Rozengaarde, Mark Berkum and Mark Raalterwold, Mark het Gooi and Mark Bestmen, and between Mark Bestmen and Mark Raalterwold, S2 Table). Temporal associations between pairs of commons were equally rare for bureaucratic (3 of 36 pairwise comparisons) and for resource related rule changes (3 of 36, S3 Table). An association of temporal distributions of general sanctioning was evident in only one of the 45 pairwise comparisons (between commons Mark Geesteren Mander en Vasse and Mark Berkum, *r*_s_ = 0.18, *p* = 0.0027, *n* = 275 years). The results and conclusions remained unchanged when the same associations were checked using the maximal information criterion [36] to explore more complex (e.g., periodic) relationships between commons in terms of rule changes, revealing no strong similarities between pairs of commons (none exceeding 0.1, for details see S2b and S3b Tables).

A possible reason for the general lack of synchrony, albeit with a few exceptions, is that the different commons were organized to manage the use of different combinations of shared goods, such that specific solutions and adaptations were required depending on environmental challenges, socioeconomic conditions, and external pressures. In keeping with this notion, we hypothesized that pairs of commons that utilized similar resources likely were influenced by similar environmental, political and socioeconomic challenges and circumstances, and they were therefore predicted to have more synchronized temporal activity patterns compared with pairs of commons that were built around different resources. This prediction was not supported by the data; pairwise differences between commons in temporal distributions of regulatory activities were not associated with pairwise differences in resource composition (matrix correlation, *r* = 0.028, *n* = 36, the 95% confidence interval based on 500 permutations was -0.079 to 0.26) (S2 Fig). A Monte-Carlo test generated a similar result (*r* = 0.06, *p* = 0.39). Our present results for Dutch commons thus do not conform to patterns documented in biological systems of convergent evolution and more synchronous abundance fluctuations in species that occupy similar niches and environments [22–25,39].

We cannot identify with certainty based on available data why the institutional developments were not more similar overall between commons that used more similar resources. However, the time over which data on institutional change was recorded span more than 600 years, and although there were time windows when the different commons coexisted, there were also differences both with regards to the year of origination and the lifespan of commons (Fig 2). This means that there were periods when the commons experienced different legislative culture, political and socioeconomic challenges, and environmental conditions (for details see S9 and S10 Tables). General circumstances and external drivers may therefore have called for different solutions even by commons that used similar resources. In general, the rate of institutional change (i.e., when the written regulations were established, modified, replaced or abandoned) varied remarkably over time (Fig 2). However, our data suggests that times of intense regulatory activities were not strongly correlated with or restricted to periods with external challenges (compare Fig 2 and S9 and S10 Tables), unlike biological systems where changing environmental conditions can induce micro-evolutionary shifts and genetic rearrangements [23,28]. This indicates that the evolution of institutions in the historical commons studied here was probably influenced by several interacting external and internal factors. It is particularly noticeable that the increasing resource demand associated with the rapid increase in human population size and density in our study area during ca 800–1850 (see Fig 4 in ref [15]), was not accompanied by a parallel consistent increase in the rate of institutional evolution. It is possible, however, that temporary changes in management and resource use occurred without leaving any traces in the written regulations as described in the institutions, comparable to development plasticity and phenotypic flexibility in biological species [31–33].

That the temporal distributions of regulatory activities were not correlated between commons may also be reflective of internal factors, such as succession of commoners and decision makers with different philosophies, ideas and convictions regarding best practice institution management. If decision making within commons was influenced by the composition of commoners and by their relatedness, reputation, and experiences, this likely contributed a stochastic dimension to the development of the regulatory activities. This would resemble to some extent the contribution to genetic variation in natural populations of biological species by random events such as mutation and drift.

On a more technical note, the periods of pairwise temporal coexistence used for the computations of the distance matrices may have covered different parts of the life of the commons, and the strength of the association may therefore have been underestimated. Although the results so far indicate that institutional developments have been largely independent and not clearly related with resource use, our findings do point to an interesting role of sanctioning regulations, reported below.

### On the role of sanctioning for non-renewable resources and lifespan of commons

Social dilemmas, conflicts of interest and selfish, short-term temptations may lead to overuse and ultimately depletion of non-renewable resources unless appropriately managed and regulated. Accordingly, systems of rules and enforcement mechanisms have been considered key to prevent overuse and achieve collectively beneficial outcomes, including both welfare of the commoners and sustainability of the resource use [3–7,9–12,40]. However, the role of sanctioning for the success of commons has received mixed empirical support. For example, Farjam, de Moor [9] report that the incidence of sanctioning declined over time across commons in the Netherlands, but not in the UK. A possible explanation for these discrepancies might be that the importance of enforcement mechanisms is not independent of the nature of the shared goods. Instead, it can be hypothesized that sanctioning and enforcement are more important in preventing overuse of non-renewable resources. If so, the incidence of sanctioning should vary among commons according to the types of shared resources, and sanctioning should play a more prominent role in commons that rely more heavily on finite resources.

**Fig 3:**
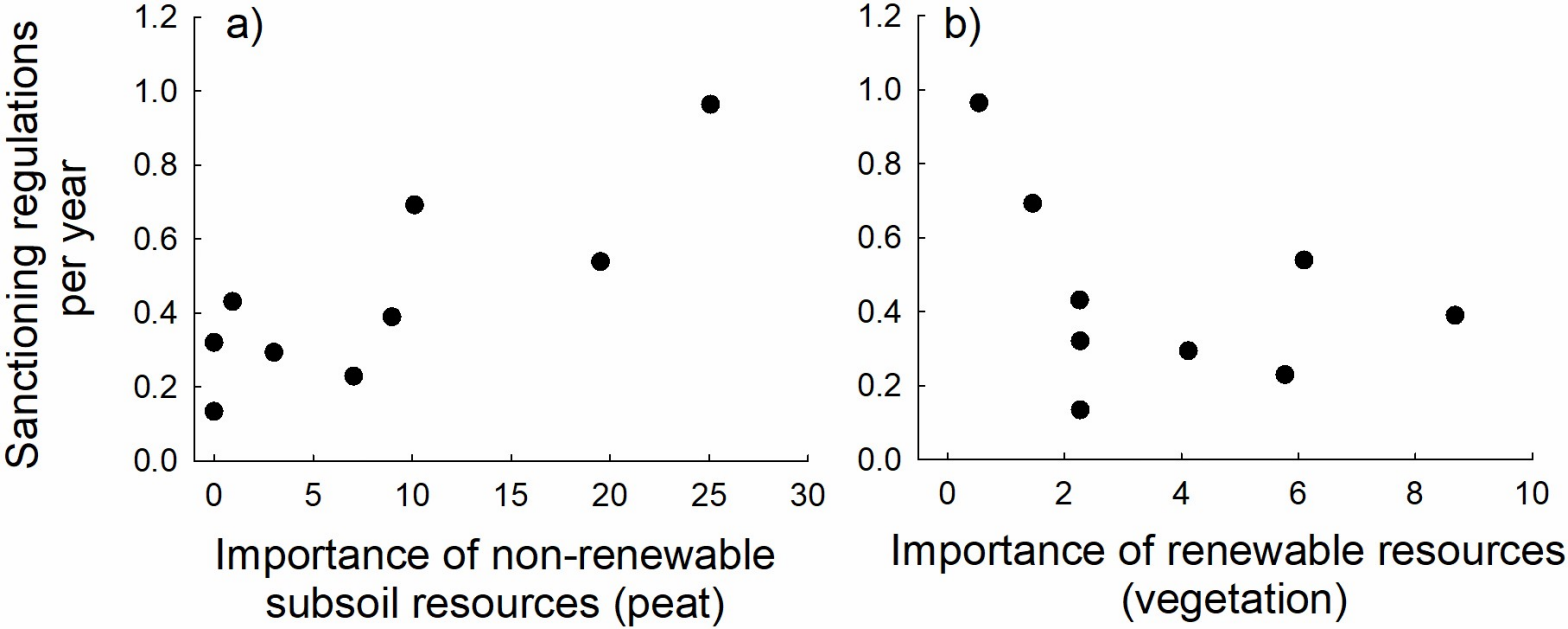
Relationship across Dutch commons between the annual frequencies of enforcement mechanisms in the form of sanctioning related regulations and the relative amount of sanctioning regulations directed to a) finite subsoil (primarily peat) resources *r* = 0.82, *N* = 9, *P* = 0.0066 and b) renewable (vegetation) resources *r* = -0.39, *N* = 9, *P* = 0.30.

Our results were seemingly consistent with the above hypothesis; across commons the number of sanctioning regulations per year increased with increasing importance of (as indicated by the amount of management directed towards) non-renewable subsoil resources, such as peat (*r* = 0.82, *n* = 9, *p* = 0.0066) (Fig 3a). However, the variation in the rate of sanctioning regulations among commons was not associated with the use of renewable resources in the form of vegetation, and the non-significant trend was negative (not positive as for non-renewable resources) (*r* = -0.39, *n* = 9, *p* = 0.29, Fig 3b). Together, these relationships might indicate that commoners were aware that non-renewable resources required more formal regulation and restricted utilization. This is in accordance with Ostrom’s [1,2,10] notion that rules and graduated sanctioning may promote well-functioning commons and sustainable utilization under social dilemmas. Indeed, short-term and sometimes selfish interests may result in behaviors of individuals as consumers and of companies as providers of resources that are potentially in conflict with the long-term benefits of society as a whole [41].

The role of sanctioning for sustainable resource use and viability of commons is, however, uncertain and conclusions must be tentative. However, preliminary results based on data for the nine Dutch commons included in this study indicate that life span decreased with increasing use of sanctioning (*r* = -0.74, *n* = 9, *p* = 0.023, see also De Moor and Tukker [34]). Results from higher resolution analyses based on data for a larger data set that comprises historical commons in the Netherlands, United Kingdom and Spain may add a layer of generality to this issue and inform whether a sizeable fraction of the variance in lifespan of commons can be generally accounted for by differential sanctioning intensity or whether the consequences of sanctioning are context specific (De Moor et al. *in preparation*). The pattern reported here nevertheless raises the question whether sanctioning regulations were imposed more frequently towards the end of the life of commons as an act of desperation to solve problems associated with overexploitation. Our analyses suggests that this was not the case. In general, the rate of sanctioning in these Dutch commons was higher during the first than during second half of the life of commons (first half, mean ± std: 0.53 ± 0.242 sanctioning related regulations/year; second half: 0.34 ± 0.208, paired comparisons *t*-test, = 3.30, *df* = 8, *p* = 0.0109). The higher rate of sanctioning during the first half of the life of commons was even more pronounced when expressed as a fraction of all regulatory activities (first half, mean ± std: 0.55 ± 0.124; second half: 0.26 ± 0.159, *t* = 7.90, *df* = 8, *p* < 0.0001). Together, this seems to suggest that sanctioning might have been important during the establishment of commons, and further that commons were dissolved not because but despite sanctioning related regulations.

In analogy with biological sciences, our analyses of historical commons resembles in some ways attempts to reconstruct the dynamics of extinct species based on an incomplete fossil record. Admittedly, the number of commons used for these analyses is limited and correlational approaches, such as ours, cannot inform about causality. Several external factors including social, political and economic pressures, new reclamations of resources and pressure on resources due to population growth, together with environmental conditions such as natural disasters (S9 and S10 Tables) and climate change may contribute to variation in lifespan and the dissolution of commons [15,34,42]. The majority of Dutch commons was dissolved during the 19^th^ century with a clear peak around 1850 [15], partly as the result of the increasingly stringent legislation and increasing taxation that was putting pressure on commoners to privatize their commons [43–45].

It remains uncertain whether the higher rate of sanctioning in commons that used non-renewable resources to a higher degree (Fig 3a) was imposed specifically to prevent the overuse of depletable resources, or whether the sanctioning instead aimed to resolve conflicts associated with competing demands and alternative land use. Areas that were harvested for subsoil resources such as peat were not simultaneously available for alternative use, such as grazing livestock. On the other hand, previous results based on analysis of a larger data set suggest that commons that relied on more resource types survived for longer [15]. This might indicate that costs associated with competing needs were counterbalanced by benefits associated with the variance reducing portfolio effect that comes with generalism in a broad range of biological systems, commercial companies, and service providing organizations [46–53]. However, to evaluate whether sanctioning regulations were primarily imposed to avoid overexploitation and/or to govern alternative land use will require more in-depth analyses of the dynamics of regulatory activities. There is also a need to evaluate whether there are specific types of sanctioning that are more effective than others in achieving sustainable utilization of resources and long-term resilience of commons [1].

## Conclusions

Our findings provide new knowledge of the evolution and dynamics of commons as for the management and use of collective resources. The overall asynchrony, independent long-term developments, and lack of association between resource use and the temporal distribution of regulatory activities together point to a complex interplay of internal properties and external factors as drivers of institutional change. Perhaps the most interesting and novel finding, and one striking exception to the overall context specificity, was that enforcement mechanisms were more abundant in commons that depended more on non-renewable subsoil resources (peat), a pattern that may be reflective of a desire to avoid overexploitation or to resolve conflicts associated with competing land use. Further in-depth and higher resolution analysis is required (and underway) to evaluate whether sanctioning offered an effective means of increasing viability of the historical commons studied here. That sanctioning related regulations were more frequent during the early than during the late part of the commons life nevertheless suggests that enforcements were important during the establishment phase. Conversely, sanctions were not a major driver of extinction, and nor does it seem as if sanctions were used as (unsuccessful) attempts to circumvent problems that were leading to extinction. As such, our study exemplifies how interdisciplinary approaches can further understanding of the long-term development of historical commons institutions, and has policy relevance for the currently developing new wave of institutions for collective action [54].

### Speculation

Our present findings provide mixed support for the notion based on results from previous analyses that the cultural evolution exhibited by commons institutions (with regards to their emergence, dispersion, and dissolution) follow patterns that are similar to those detected in biological populations, species and communities [15]. Eco-evolutionary theory and empirical evidence concur that species that utilize comparable resources and environments should converge on a shared evolutionary solution [22,23] and show correlated population dynamic responses [24,25,39], whereas species that occupy different niches respond independently. Our present findings indicate that historical commons that used more similar resources did not have more parallel or similar institutional developments, with regards to the temporal distribution of regulatory activities.

In analogy with biological systems, the occasional periods of intense clumped regulatory activity seen in some of the commons (Fig 2) might be interpreted as evolutionary adaptations during times with rapid and drastic shifts in environmental conditions and selection regimes [26–28]. In keeping with notion, the extended periods of low activity seen in some commons might be thought of as representing a lack of evolutionary modifications (evolutionary stasis) expected in constant environments that impose stabilizing selection [29]. However, this interpretation is difficult to reconcile with the temporal distribution of environmental, political, socioeconomic, and legislative pressures that the commons were subjected to throughout history (S9 and S10 Tables). Although many of these pressures undoubtedly changed the demand, availability, utilization and benefits of the shared resources, they did not coincide on an overall level with the periods of intensified regulatory activity. This seems to suggest that the temporal distribution of institutional changes that were recorded in the commons studied here does not share any strong resemblance to the rapid evolutionary adaptations observed in biological species in response to strong directional selection [23,28]. It also seems unlikely that the reoccurring and extended periods of inactivity (with regards to regulatory activities) seen in some commons, and the continuously low activity seen in other commons (Fig 2), reflect stable internal and external environmental conditions that did not require any modifications of management and resource use. An alternative interpretation is that the behaviors and utilization patterns of the commoners changed over time also during these periods, but without institutional alterations of the written rules concerning the use, governance and management of resources. This would be analogous to phenotypic flexibility and developmental plasticity that can manifest in biological systems in the absence of changes in the genetic code [31–33]. One possibility worth investigating is whether changes in management preceded changes in the formal institutions, analogous to genetic assimilation (‘genes as followers’) in biological evolution where phenotypic change initially results from plastic responses to environmental influences that are subsequently incorporated into the genome [55].

Continuing along the path of speculations, it might be argued that the decision making and system of rules used in the historical commons studied here might inform management and use of shared resources in contemporary and future social-ecological systems. As reported here, and earlier [15], many of the historical Dutch commons were remarkably long-lived and survived for several centuries. Contrary to other, typically shorter-lived organizations that are private (and thus run by a limited number of individuals having an impact on decision making and the course of the organization) or public (whereby the state coordinates the changes and evolution of the organization), commons depend highly on self-organization and cooperation by most members of the group in order to ensure the organization can survive. This suggests that looking back into the past can help us find ways forward towards increased resilience and advice about more sustainable utilization of shared resources.

#### Ethics statement

This article does not present research with ethical considerations.

## Supporting information

S1 Fig Relationship between bureaucratic, resource related and sanctioning regulatory activities (rules changes). Figure shows results based on analyses of pooled data for nine Dutch commons. Statistical results represent Spearman correlation coefficients, all *n* = 3329.
(PDF)

S2 Fig Relationship across Dutch commons between pairwise distances in temporal distribution of regulatory activities and pairwise distances in the composition of resources used. (PDF)

S1 Table Characterization of nine historical Dutch commons according to the number of regulatory activities that pertain to different types of resources. This is a higher resolution version of the information on resource use provided in Table 1.
(PDF)

S2 Table Pairwise associations of temporal distributions of regulatory activities between nine Dutch commons. Correlation matrix shows results from: **a)** Spearman rank correlation analyses (*r*_s_, *P*, and *n*-values) between pairs of commons. * indicates that the association was statistically significant after Bonferroni correction (α, 0.05/36 = 0.0014). **b)** Pairwise maximal information (according to [36]). See Fig 2 or Table 1 for a key to Common IDs.
(PDF)

S3 Table Associations between temporal distributions of bureaucratic related rules changes (below the diagonal) and resource related rules changes (above the diagonal) among nine Dutch commons. Correlation matrix shows results from: **a)** Spearman rank correlation analyses (*r*_s_, *P*, and *n*-values) between pairs of commons. * indicates that the association was statistically significant after Bonferroni correction (α, 0.05/36 = 0.0014). **b)** Pairwise maximal information (according to [36]).
(PDF)

S4 Table Loadings (eigenvectors) of resources on the first four principal components derived from a correlation matrix of eight types of resources (Table 1) based on data for historical Dutch commons. (PDF)

S5 Table Pairwise distance (Eucilidan) matrix between Dutch commons based on data on eight types of resources. See Table 1 for a key to Common IDs.
(PDF)

S6 Table Pairwise distance (Eucilidan) matrix between Dutch commons based on ordinal data on number of yearly regulatory activities (rule changes). See Table 1 for a key to Common IDs.
(PDF)

S7 Table Comparisons (goodness of fit statistics) of Poisson regression models in which count data on number of regulatory activities per year was treated as response variable, common identity was treated as a fixed class variable, and linear (Y), quadratic (Y2) and cubic (Y3) effects of calendar year were treated as continuous predictor variables. Models were fitted using procedure GENMOD in SAS. The response variable was modeled with a zero inflated negative binomial (ZINB) distribution. The Pearson Chi-square statistic and associated p-value constitute formal tests for overdispersion and indicate that the null hypothesis of no overdispersion is not rejected for any model. Deviance is a measure of goodness-of-fit for the model. The Akaike information criterion (AIC, smaller is better) estimates the relative quality of statistical models for a given set of data.
(PDF)

S8 Table Analyses restricted to the six commons for which longitudinal times series data is available for at least ≥249 years (i.e., common IDs 15, 113, 149, 380, 395 and 440, see Fig 2). Comparisons (goodness of fit statistics) of Poisson regression models in which count data on number of regulatory activities per year was treated as response variable, common identity was treated as a fixed class variable, and linear (Y), quadratic (Y^2^) and cubic (Y^3^) effects of calendar year were treated as continuous predictor variables. Models were fitted using procedure GENMOD in SAS, and because the count data had a high incidence of zeros the response variable was modelled with a zero inflated negative binomial (ZINB) distributions. The Pearson Chi-square statistics and associated *p*-values constitute formal tests for overdispersion and indicate that the null hypothesis of no overdispersion is not rejected for any model. The Akaike information criterion (AIC, smaller is better) estimates the relative quality of statistical models for a given set of data.
(PDF)

S9 Table Legislation regarding division of Dutch commons. (PDF)

S10 Table Overview of political, socio-economical and environmental threats to Dutch commons between the 8th and the middle of the 19th century. (PDF)
