# Supplementary material for: Comparisons of historical Dutch commons inform about the long-term dynamics of social-ecological systems
Source: PLoS One. 2021 Aug 27;16(8):e0256803. doi: 10.1371/journal.pone.0256803 (PMC8396728; doi:10.1371/journal.pone.0256803)
Supplement: S1 Fig — Figure shows results based on analyses of pooled data for nine Dutch commons. Statistical results represent Spearman correlation coefficients, all n = 3329. (PDF) [file pone.0256803.s001.pdf]

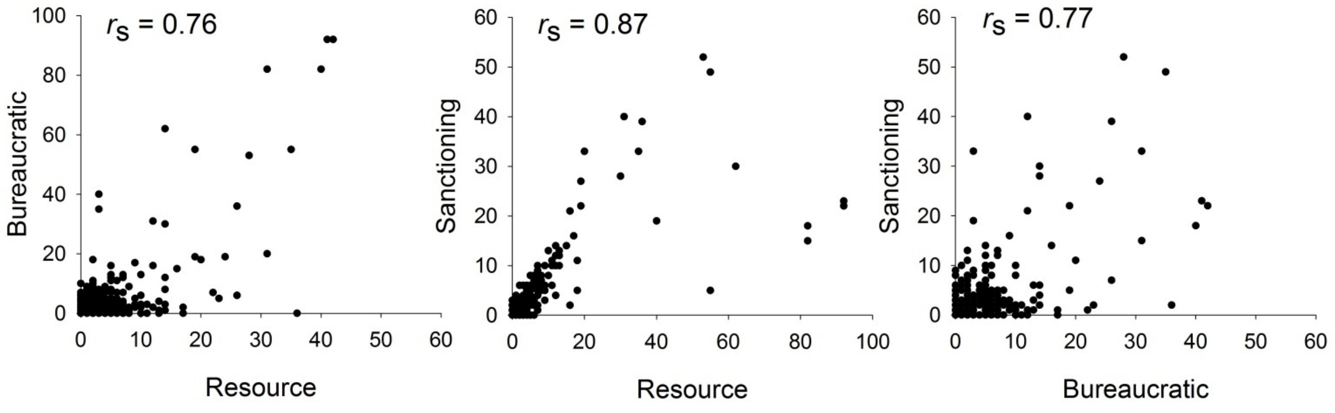

S1 Figure. Relationship between bureaucratic, resource related and sanctioning regulatory activities (rules changes). Figure shows results based on analyses of pooled data for nine Dutch commons. Statistical results represent Spearman correlation coefficients, all *n* = 3329.
