# Supplementary material for: Comparisons of historical Dutch commons inform about the long-term dynamics of social-ecological systems
Source: PLoS One. 2021 Aug 27;16(8):e0256803. doi: 10.1371/journal.pone.0256803 (PMC8396728; doi:10.1371/journal.pone.0256803)
Supplement: S2 Fig — (PDF) [file pone.0256803.s002.pdf]

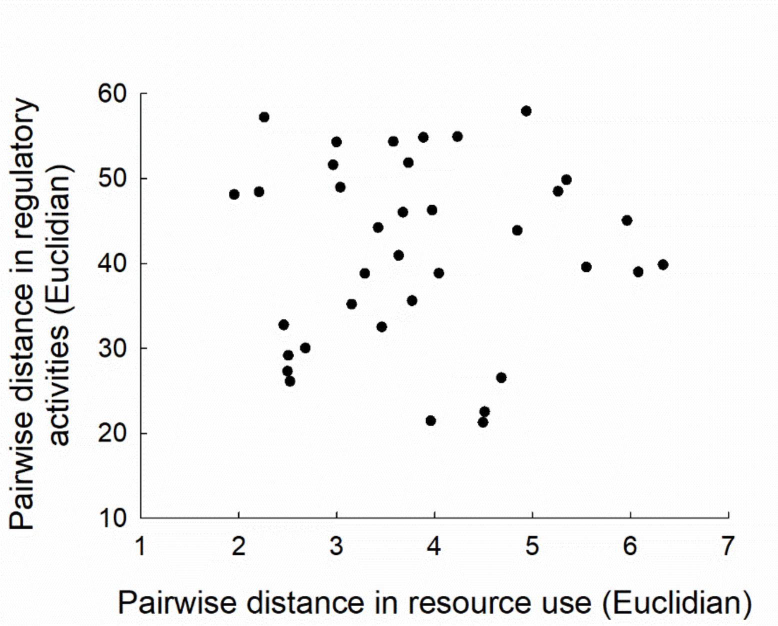

S2 Figure. Relationship across Dutch commons between pairwise distances in temporal distribution of regulatory activities and pairwise distances in the composition of resources used.
