# Supplementary material for: Comparisons of historical Dutch commons inform about the long-term dynamics of social-ecological systems
Source: PLoS One. 2021 Aug 27;16(8):e0256803. doi: 10.1371/journal.pone.0256803 (PMC8396728; doi:10.1371/journal.pone.0256803)
Supplement: S1 Table — This is a higher resolution version of the information on resource use provided in Table 1. (PDF) [file pone.0256803.s003.pdf]

S1 Table. Characterization of nine historical Dutch commons according to the number of regulatory activities that pertain to different types of resources. This is a higher resolution version of the information on resource use provided in **Table 1**.

**Type of resource**	Mark Exel	Mark Raalterwold	Mark Berkum	Mark Bestmen	Mark Coevorden	Dunsborger Hattemer mark	Mark Geesteren, Mander en Vasse	Mark het Gooi	Mark Rozengaarde
**Animals**									
Cows	4	16	2	9	1		10	137	12
Geese	7	27	9	1	3			5	1
Horses	5	26	3	6	15	2	7	111	13
Pigs	9	16	7	2	2	2	5	8	7
Sheep	27	44	6	36	4	14	28	51	6
Unspecified	22	49	31	10	40	20	27	201	58
Fish			6						
Bees				1		8			
Oxen								8	1
Other									2
Animals Total	74	178	64	65	65	46	77	521	100
**Borders**									
Conflicts		3	1				7		11
Fences		1	6	9	4	1	3	1	6
Setting of Borders	9	3	1			3	8	5	3
Right of Way									2
Borders Total	9	7	8	9	4	4	18	6	22
**Housing**									
Cabins	18				3	17	4		2
General	1	10			19		8		
Manorial Farms		1							
Other		6			1		3		

Peasant Farms		14							
Housing Total	19	31			23	17	15		2
**Infrastructure**									
Bridges	1	11		1	5		2		4
Culverts and Sluices (Drainage)		4	1						3
Ditches (Drainage)	8	4	7		9				2
Dykes	6	3	35		10			1	18
General		2						4	
Leat (Drainage)	5	37				1			
Other		1	2						2
Roads		5	6			5			4
Waterways (Drainage) General		1	5	2		8	4	4	2
Infrastructure Total	20	68	56	3	24	14	6	9	35
**Subsoil Resources**									
Clay		4		1					
Loam	1	4		1				3	
Peat	92	68		9	2	48	30	7	
Dredgings								12	
Subsoil Resources Total	93	76		11	2	48	30	22	
**Topsoil Resources**									
(Drifting) Sand		8		13		10		2	
Diggings general	18	54	4		4	13	5	4	5
Land		62	8	1	1	6	7	8	13
Manure	1	6							
Sods	17	17		8	2	8	16	9	3
Toppeat (schelturf)		10				2			
Stones								4	
Topsoil Resources Total	36	157	12	22	7	39	28	27	21
Animals - Unspecified		2							
Unspecified	118	221	75	37	82	63	131	114	78

Unspecified Total	118	223	75	37	82	63	131	114	78
**Vegetation**									
Crops (in general)		1				2	1		1
Grass		3					1	4	2
Hay	1	3	4				1		1
Heath		2		1		5	1	7	
Moss		1							
Other		1						1	
Reeds	1							3	
Wood				7	5	8	24	2	2
Acorns				1					
USPVEGSTB			1						
Stalks							1		
Rushes								6	
Thistle								1	
Sedges								6	
Vegetation Total	2	11	5	9	5	15	29	30	6
**Grand Total**	371	751	220	156	212	246	334	729	264
