# Supplementary material for: Comparisons of historical Dutch commons inform about the long-term dynamics of social-ecological systems
Source: PLoS One. 2021 Aug 27;16(8):e0256803. doi: 10.1371/journal.pone.0256803 (PMC8396728; doi:10.1371/journal.pone.0256803)
Supplement: S2 Table — Correlation matrix shows results from: a) Spearman rank correlation analyses (rs, P, and n-values) between pairs of commons. * indicates that the association was statistically significant after Bonferroni correction (α, 0.05/36 = 0.0014). b) Pairwise maximal information (according to [36]). See Fig 2 or Table 1 for a key to Common IDs. (PDF) [file pone.0256803.s004.pdf]

S2 Table. Pairwise associations of temporal distributions of regulatory activities between nine Dutch commons. Correlation matrix shows results from: **a)** Spearman rank correlation analyses (r_s, P, and n-values) between pairs of commons. * indicates that the association was statistically significant after Bonferroni correction (α, 0.05/36 = 0.0014). **b)** Pairwise maximal information (according to [1]).
See **Figure 2** or **Table 1** for a key to Common IDs.

a)

	**15**	**113**	**149**	**179**	**231**	**251**	**380**	**395**	**440**
**15**									
**113**	0.07836<br>0.1951<br>275								
**149**	0.04308<br>0.4768<br>275	-0.03295<br>0.4971<br>427							
**179**	0.01398<br>0.8620<br>157	0.01835<br>0.7890<br>215	0.01080<br>0.8734<br>220						
**231**	-0.04845<br>0.4746<br>220	-0.01224<br>0.8476<br>249	-0.00910<br>0.8864<br>249	0.08958<br>0.2240<br>186					
**251**	-0.08517<br>0.2001<br>228	0.03403<br>0.6076<br>230	-0.02182<br>0.7421<br>230	0.00873<br>0.9131<br>159	-0.00225<br>0.9734<br>222				
**380**	-0.01606<br>0.7909<br>275	0.03002<br>0.5633<br>373	**0.16329***<br>**0.0013**<br>**385**	0.06809<br>0.3147<br>220	-0.06048<br>0.3419<br>249	0.05982<br>0.3665<br>230			
**395**	**0.25549***<br>**<.0001**<br>**273**	0.01907<br>0.7460<br>291	0.09628<br>0.1012<br>291	-0.08621<br>0.2861<br>155	-0.08283<br>0.2232<br>218	-0.05860<br>0.3806<br>226	-0.00069<br>0.9906<br>291		
**440**	0.00850<br>0.8885<br>275	**0.13203**<br>**0.0094**<br>**386**	0.03467<br>0.4898<br>399	-0.00725<br>0.9148<br>220	-0.06996<br>0.2714<br>249	-0.04446<br>0.5023<br>230	**0.13738**<br>**0.0069**<br>**385**	0.00526<br>0.9288<br>291	

b)

	**15**	**113**	**149**	**179**	**231**	**251**	**380**	**395**	**440**
**15**									
**113**	0.0088								
**149**	0.02643	0.00112							
**179**	0.05029	0.04496	0.02835						
**231**	0.05152	0.04267	0.01593	0.11913					
**251**	0.00287	0.04382	0.00037	0.03119	0.02938				
**380**	0.03122	0.05823	0.00138	0.07959	0.05007	0.028			
**395**	0.07263	0.03108	0.04332	0.03556	0.0474	0.00169	0.05622		
**440**	0.08446	0.06605	0.02561	0.07598	0.09373	0.03203	0.07317	0.08634	

1. Reshef DN, Reshef YA, Finucane HK, Grossman SR, McVean G, Turnbaugh PJ, et al. Detecting Novel Associations in Large Data Sets. Science. 2011;334(6062):1518-24. doi: 10.1126/science.1205438.
