# Supplementary material for: Comparisons of historical Dutch commons inform about the long-term dynamics of social-ecological systems
Source: PLoS One. 2021 Aug 27;16(8):e0256803. doi: 10.1371/journal.pone.0256803 (PMC8396728; doi:10.1371/journal.pone.0256803)
Supplement: S3 Table — Correlation matrix shows results from: a) Spearman rank correlation analyses (rs, P, and n-values) between pairs of commons. * indicates that the association was statistically significant after Bonferroni correction (α, 0.05/36 = 0.0014). b) Pairwise maximal information (according to [36]). (PDF) [file pone.0256803.s005.pdf]

S3 Table. Associations between temporal distributions of bureaucratic related rules changes (below the diagonal) and resource related rules changes (above the diagonal) among nine Dutch commons. Correlation matrix shows results from: **a)** Spearman rank correlation analyses (r_s, *P*, and *n*-values) between pairs of commons. * indicates that the association was statistically significant after Bonferroni correction (α, 0.05/36 = 0.0014). **b)** Pairwise maximal information (according to [1]).

a)

	15	113	149	179	231	251	380	395	440
		**0.13013**	0.06665	0.05572	-0.06022	-0.07164	-0.02596	**0.26109***	0.02203
**15**		**0.0310**	0.2707	0.4883	0.3741	0.2814	0.6682	**<.0001**	0.7161
		**275**	275	157	220	228	275	**273**	275
	0.09547		-0.02543	-0.00204	-0.04365	0.05534	0.01788	0.06804	**0.20834***
**113**	0.1142		0.6002	0.9763	0.4929	0.4035	0.7308	0.2473	**<.0001**
	275		427	215	249	230	373	291	**386**
	0.04387	-0.02898		0.01418	-0.00418	-0.02182	0.07865	0.11113	0.05046
**149**	0.4687	0.5504		0.8343	0.9477	0.7421	0.1234	0.0583	0.3147
	275	427		220	249	230	385	291	399
	0.02220	0.01303	0.03284		0.11640	0.02399	0.07614	-0.11823	0.04520
**179**	0.7825	0.8494	0.6281		0.1136	0.7641	0.2608	0.1429	0.5048
	157	215	220		186	159	220	155	220
	0.03114	0.00444	0.01350	0.12110		0.01407	-0.10612	-0.05146	-0.08440
**231**	0.6460	0.9444	0.8321	0.0997		0.8349	0.0947	0.4497	0.1843
	220	249	249	186		222	249	218	249
	-0.07312	0.06339	-0.02036	0.00307	-0.01556		0.07584	-0.04930	-0.02912
**251**	0.2716	0.3386	0.7587	0.9694	0.8177		0.2520	0.4608	0.6604
	228	230	230	159	222		230	226	230
	0.02096	0.07157	**0.19639***	0.07279	-0.06903	0.07990		0.02536	0.07700
**380**	0.7293	0.1678	**0.0001**	0.2824	0.2779	0.2274		0.6666	0.1315
	275	373	**385**	220	249	230		291	385
	**0.26335***	-0.00517	0.10349	-0.13264	-0.05507	-0.05138	0.01892		-0.00624
**395**	**<.0001**	0.9300	0.0780	0.0999	0.4185	0.4421	0.7479		0.9156
	**273**	291	291	155	218	226	291		291
	-0.00851	0.01332	0.03646	-0.00654	-0.01053	-0.03221	**0.15525**	0.04146	
**440**	0.8883	0.7941	0.4677	0.9232	0.8687	0.6270	**0.0023**	0.4811	
	275	386	399	220	249	230	**385**	291	

b)

	15	113	149	179	231	251	380	395	440
**15**		0.00251	0.04375	0.0339	0.03461	0.00149	0.01928	0.04659	0.08534
**113**	0.00632		0.00061	0.02244	0.01715	0.05622	0.031	0.03393	0.07967
**149**	0.02637	0.00087		0.03282	0.00755	0.00037	0.00125	0.03079	0.02803
**179**	0.04999	0.0153	0.02236		0.08191	0.04344	0.07101	0.01768	0.0995
**231**	0.07775	0.03107	0.0161	0.05525		0.02575	0.02909	0.01992	0.082
**251**	0.00267	0.04344	0.00037	0.02032	0.01852		0.04332	0.0013	0.03845
**380**	0.01675	0.04071	0.00112	0.06567	0.01757	0.03091		0.04332	0.06903
**395**	0.07837	0.00399	0.04332	0.02218	0.0444	0.00169	0.03528		0.02221
**440**	0.06971	0.01846	0.01533	0.04531	0.04614	0.02009	0.06565	0.06372	

1. Reshef DN, Reshef YA, Finucane HK, Grossman SR, McVean G, Turnbaugh PJ, et al. Detecting Novel Associations in Large Data Sets. Science. 2011;334(6062):1518-24. doi: 10.1126/science.1205438.
