# Supplementary material for: Comparisons of historical Dutch commons inform about the long-term dynamics of social-ecological systems
Source: PLoS One. 2021 Aug 27;16(8):e0256803. doi: 10.1371/journal.pone.0256803 (PMC8396728; doi:10.1371/journal.pone.0256803)
Supplement: S4 Table — (PDF) [file pone.0256803.s006.pdf]

S4 Table. Loadings (eigenvectors) of resources on the first four principal components derived from a correlation matrix of eight types of resources (**Table 1**) based on data for historical Dutch commons.

	**Eigenvectors**			
**Resource**	**Prin1**	**Prin2**	**Prin3**	**Prin4**
Animals	0.025877	0.636303	0.031457	-.412991
Borders	-.141121	-.022976	0.763056	0.530010
Housing	0.447329	-.134515	-.255963	0.285363
Infrastructure	0.327611	-.276223	0.428572	-.530722
Subsoil	0.420500	0.039285	-.280283	0.361160
Topsoil	0.503686	0.075039	0.151043	-.063993
Vegetation	-.045725	0.667130	0.038105	0.221009
Unspecified	0.489258	0.218999	0.254441	0.045317
Eigenvalue	3.34	1.87	1.12	0.93
Variance explained	41.8%	23.3%	14.0%	11.6%
