# Supplementary material for: Comparisons of historical Dutch commons inform about the long-term dynamics of social-ecological systems
Source: PLoS One. 2021 Aug 27;16(8):e0256803. doi: 10.1371/journal.pone.0256803 (PMC8396728; doi:10.1371/journal.pone.0256803)
Supplement: S5 Table — See Table 1 for a key to Common IDs. (PDF) [file pone.0256803.s007.pdf]

S5 Table. Pairwise distance (Eucilidan) matrix between Dutch commons based on data on eight types of resources. See **Table 1** for a key to Common IDs.

**Common ID**	**15**	**113**	**149**	**179**	**231**	**251**	**380**	**395**	**440**
**15**	0.00000	.	.	.	.	.	.	.	.
**113**	4.04335	0.00000	.	.	.	.	.	.	.
**149**	3.76966	4.51444	0.00000	.	.	.	.	.	.
**179**	3.57768	3.67669	4.84562	0.00000	.	.	.	.	.
**231**	2.99782	3.03736	3.97549	2.25993	0.00000	.	.	.	.
**251**	3.63201	2.50577	4.49593	2.96249	1.95343	0.00000	.	.	.
**380**	3.28816	2.49787	3.95975	3.42245	2.20720	2.52470	0.00000	.	.
**395**	3.15288	2.45896	4.68430	3.88556	3.73165	3.46089	2.68010	0.00000	.
**440**	5.34727	5.54954	6.08153	4.23337	4.93677	5.26044	6.33413	5.96633	0
