# Supplementary material for: Comparisons of historical Dutch commons inform about the long-term dynamics of social-ecological systems
Source: PLoS One. 2021 Aug 27;16(8):e0256803. doi: 10.1371/journal.pone.0256803 (PMC8396728; doi:10.1371/journal.pone.0256803)
Supplement: S6 Table — See Table 1 for a key to Common IDs. (PDF) [file pone.0256803.s008.pdf]

S6 Table. Pairwise distance (Eucilidan) matrix between Dutch commons based on ordinal data on number of yearly regulatory activities (rule changes). See **Table 1** for a key to Common IDs.

Common ID	15	113	149	179	231	251	380	395	440
**15**	0.0000	.	.	.	.	.	.	.	.
**113**	38.8381	0.0000	.	.	.	.	.	.	.
**149**	35.6034	22.5448	0.0000	.	.	.	.	.	.
**179**	54.3303	46.0177	43.8805	0.0000	.	.	.	.	.
**231**	54.3047	48.9685	46.2831	57.2135	0.0000	.	.	.	.
**251**	40.9514	29.1607	21.2930	51.6026	48.1037	0.0000	.	.	.
**380**	38.8072	27.3151	21.4742	44.2210	48.4196	26.1151	0.0000	.	.
**395**	35.2110	32.7591	26.5586	54.8417	51.8453	32.5216	30.0258	0.0000	.
**440**	49.8357	39.5539	38.9926	54.9318	57.9319	48.4885	39.8318	45.0460	0
