# Supplementary material for: Comparisons of historical Dutch commons inform about the long-term dynamics of social-ecological systems
Source: PLoS One. 2021 Aug 27;16(8):e0256803. doi: 10.1371/journal.pone.0256803 (PMC8396728; doi:10.1371/journal.pone.0256803)
Supplement: S7 Table — Models were fitted using procedure GENMOD in SAS. The response variable was modeled with a zero inflated negative binomial (ZINB) distribution. The Pearson Chi-square statistic and associated p-value constitute formal tests for overdispersion and indicate that the null hypothesis of no overdispersion is not rejected for any model. Deviance is a measure of goodness-of-fit for the model. The Akaike information criterion (AIC, smaller is better) estimates the relative quality of statistical models for a given set of data. (PDF) [file pone.0256803.s009.pdf]

S7 Table. Comparisons (goodness of fit statistics) of Poisson regression models in which count data on number of regulatory activities per year was treated as response variable, common identity was treated as a fixed class variable, and linear (Y), quadratic (Y^2) and cubic (Y^3) effects of calendar year were treated as continuous predictor variables. Models were fitted using procedure GENMOD in SAS. The response variable was modeled with a zero inflated negative binomial (ZINB) distribution. The Pearson Chi-square statistic and associated *p*-value constitute formal tests for overdispersion and indicate that the null hypothesis of no overdispersion is not rejected for any model. Deviance is a measure of goodness-of-fit for the model. The Akaike information criterion (AIC, smaller is better) estimates the relative quality of statistical models for a given set of data [1].

Model	Deviance	DF	Value/DF	Scaled Pearson Chi-square	*p*-value	AIC (smaller is better)
**1) C Y Y^2 Y^3 C*Y C*Y^2 C*Y^3**	3679.03	3026	0.9409	2847.16	0.99	3791.03
**2)** C Y Y^2 Y^3 C*Y C*Y^2	3698.03	3034	0.9745	2956.53	0.84	3794.03
**3)** C Y Y^2 C*Y C*Y^2	3700.36	3035	0.9576	2906.32	0.96	3794.36
**4)** C Y Y^2 C*Y	3723.15	3043	0.9600	2921.23	0.94	3801.15

Results from model comparisons based on Likelihood ratio tests (LRT):

Effect of removing $year^3$ and the interaction between common ID and $year^3$: Model 1 versus 3: $\chi^2 = 21.0$, df = 9, $0.01 < P < 0.025$

Effect of removing the interaction between common ID and $year^2$: Model 3 versus 4: $\chi^2 = 22.79$, df = 1, $P < 0.001$

Reference

1. Burnham KP, Anderson DR. Model Selection and Multimodel Inference: A Practical Information-Theoretic Approach. New York: Springer-Verlag; 2002.
