# Supplementary material for: Comparisons of historical Dutch commons inform about the long-term dynamics of social-ecological systems
Source: PLoS One. 2021 Aug 27;16(8):e0256803. doi: 10.1371/journal.pone.0256803 (PMC8396728; doi:10.1371/journal.pone.0256803)
Supplement: S8 Table — Comparisons (goodness of fit statistics) of Poisson regression models in which count data on number of regulatory activities per year was treated as response variable, common identity was treated as a fixed class variable, and linear (Y), quadratic (Y2) and cubic (Y3) effects of calendar year were treated as continuous predictor variables. Models were fitted using procedure GENMOD in SAS, and because the count data had a high incidence of zeros the response variable was modelled with a zero inflated negative binomial (ZINB) distributions. The Pearson Chi-square statistics and associated p-values constitute formal tests for overdispersion and indicate that the null hypothesis of no overdispersion is not rejected for any model. The Akaike information criterion (AIC, smaller is better) estimates the relative quality of statistical models for a given set of data. (PDF) [file pone.0256803.s010.pdf]

S8 Table. Analyses restricted to the six commons for which longitudinal times series data is available for at least ≥249 years (i.e., common IDs 15, 113, 149, 380, 395 and 440, see **Fig. 2**). Comparisons (goodness of fit statistics) of Poisson regression models in which count data on number of regulatory activities per year was treated as response variable, common identity was treated as a fixed class variable, and linear (Y), quadratic (Y^2) and cubic (Y^3) effects of calendar year were treated as continuous predictor variables. Models were fitted using procedure GENMOD in SAS, and because the count data had a high incidence of zeros the response variable was modelled with a zero inflated negative binomial (ZINB) distributions. The Pearson Chi-square statistics and associated *p*-values constitute formal tests for overdispersion and indicate that the null hypothesis of no overdispersion is not rejected for any model. The Akaike information criterion (AIC, smaller is better) estimates the relative quality of statistical models for a given set of data [1].

Model	Deviance	DF	Value/DF	Scaled Pearson Chi-square	*p*-value	AIC (smaller is better)
**1)** C Y Y^2 Y^3 C*Y C*Y^2 C*Y^3	2736.42	2356	0.99	2333.05	0.63	2790.42
**2)** C Y Y^2 Y^3 C*Y C*Y^2	2743.11	2361	1.01	2393.05	0.31	2787.11
3) **C Y Y^2 C*Y C*Y^2**	2744.69	2362	0.99	2360.94	0.50	2786.69
**4)** C Y Y^2 C*Y	2764.85	2367	1.00	2386.29	0.39	2796.85

Results from model comparisons based on Likelihood ratio tests (LRT)

Effect of removing year^3 and the interaction between common ID and year^3: Model 1 versus 3: $\chi^2 = 8.27$, df = 6, $P > 0.2$

Effect of removing the interaction between common ID and year^2: Model 3 versus 4: $\chi^2 = 20.16$, df = 5, $P < 0.002$

Reference

1. Burnham KP, Anderson DR. Model Selection and Multimodel Inference: A Practical Information-Theoretic Approach. New York: Springer-Verlag; 2002.
