# Supplementary material for: Comparisons of historical Dutch commons inform about the long-term dynamics of social-ecological systems
Source: PLoS One. 2021 Aug 27;16(8):e0256803. doi: 10.1371/journal.pone.0256803 (PMC8396728; doi:10.1371/journal.pone.0256803)
Supplement: S9 Table — (PDF) [file pone.0256803.s011.pdf]

S9 Table. Legislation regarding division of Dutch commons

Consequences of French occupation and legislation (1795-1811)
Commoners' assemblies lose part of their public-civic rights

Royal Decree of 16 April 1809 (French occupation)
Common land that has been divided and cultivated, will be exempt from land tax for the next 50 years

Royal Decree of 10 May 1810 (French occupation)
Uncultivated common land should be divided unless division is impossible or harmful. All commons are required to appoint a committee to implement the Decree. All commons should take good care of the poor users of the commons

Law on tax exemption of newly cultivated land 1812
Land tax exemption of newly cultivated land for the next 10 years

Cadastral implementation 1832
Land being measured, ownership registered, and value estimated

Royal Decree of 28 June 1837
Re-enforcing the Royal Decree of 1810, now easier because of cadastral registration

Regulation on tax exemption 1840
Supporting Royal Decree of 1837 with tax exemption for newly cultivated land

Law on tax exemption of newly cultivated land 1848
Land tax exemption of newly cultivated land for the next 20 years, after that 50% land tax exemption for the next 20 years

Directive Ministry of Finance 1848
Directive resulted in lower costs for services delivered by Cadaster and land surveyors
