# Supplementary material for: Comparisons of historical Dutch commons inform about the long-term dynamics of social-ecological systems
Source: PLoS One. 2021 Aug 27;16(8):e0256803. doi: 10.1371/journal.pone.0256803 (PMC8396728; doi:10.1371/journal.pone.0256803)
Supplement: S10 Table — (PDF) [file pone.0256803.s012.pdf]

S10 Table. Overview of political, socio-economical and environmental threats to Dutch commons between the 8th and the middle of the 19th century

8th century Start continuous occupation city of Deventer

11th century Foundation of cities of Kampen en Zwolle; parts of Overijssel (Oversticht) befell under episcopal rule of the bishop of Utrecht

Middle Ages Cities claim to be independent cities (Rijkssteden), oppose episcopal rule. Rural areas mainly controlled by lesser noblemen, often conquering parts of land from other noblemen

1456 David of Burgundy became bishop of Utrecht, strove for unification of separate parts (drostambten) of Overijssel.

1456-1526 Attempts for unification by House of Burgundy lead to increased and combined opposition of cities and noblemen against episcopal rule > increased influence of 333rulers of neighboring province of Gelre.

1475-1625 Relative de-urbanization: from 38.0 to 27.9 percent of total Overijssel population. Main changes for Twente 21.2 > 25.5 and Vollenhove 9.5 > 13.9. Salland stable at 31.3 > 32.7. Slicher van Bath 1944, p. 55 [1]

1528 Rule of Overijssel transferred from Henry II, bishop of Utrecht to civil ruler Charles V. First mentioning of current name Overijssel.

1568 Start Dutch Revolt against Spanish rule. Province Overijssel supports Spanish King.

1570 Plundering rural areas by rebellious troups (Geuzen)

1576 Overijssel supports Pacification of Ghent on condition Catholic faith remains unaffected.

1578 Conquest of city of Deventer by Dutch troups. Province of Overijssel reluctantly joins Dutch Republic.

1578-1580 Soldiers of Dutch troups do not receive (sufficient) wages and start plundering rural areas.

1580 January: Overijssel farmers unite to battle against plunderers. Battle of Raalte, killing of about 700 farmers.

June : farmers supported by Spanish troup defeat Dutch troups in Battle of Hardenbergerheide, killing about 1,600 Dutch soldiers.

1580-1597 Times of struggles and pillaging. Behavior of Dutch troups leads to large parts of rural areas remaining Catholic rather than joining Protestantism.

1585-1605 Protestantism hardly supported by population, main part of Overijssel population remains Catholic.

1597 Dutch prince Maurits finally defeats Spanish troups. Overijssel joins (Reformed) Republic.

1601-1723 Population increase; for instance, population of village of Raalte within marke Raalterwoold doubled from 235 to 470 households Slicher van Bath 1944, p. 30 [1]; proportional increase of impoverished families within Salland region (16.9 > 19.8 percent) Slicher van Bath 1944, p. 33 [1]

1601-1833 Especially change in crops: 1601 85% land, 15% meadows, in 1833: 42 and 58, Slicher van Bath 1944, p. 412 [1]

1665 First attack by troups from Münster on behalf of Holy Roman Empire. Pillaging of rural areas, mainly Twenthe.

1672 Second attack by Münster troups. Province of Overijssel is forced to denunciate alliance with Republic and to acknowledge rule of Holy Roman Empire

1674 Province of Overijssel liberated from Münster rule, readmitted to Dutch Republic under strict conditions, leading to increased power of Dutch stadhouders within Overijssel.

1675 Flood of all Saints Day (Allerheiligenvloed) – flooding of Mastenbroek polder.

1675-1795 Total population Overijssel 1675 > 79,229, 1723: 106,934, 1795: 134,104. Slicher van Bath 1944, p. 52-53 [1]

1713 Cattle plague kills about 90 percent of all cattle in the Low Countries.

1740 Very severe winter*

1750-1830 Area for agriculture multiplies 3.5. Slicher van Bath 1944, p. 407 [1]

1763 Very severe winter

1769-1770 Cattle plague, killing about 70 percent of all cattle, mainly in northern provinces.

1784 Very severe winter

1789 Very severe winter

1795 Very severe winter

1799 Very severe winter

1803 Very severe winter

1813 Very severe winter

1814 Very severe winter

1815 Rule of King William I. Increased attention to industry: metal industry and textile industry. Improvement of infrastructure, construction of transport canals.

1825 Storm surge, causing flooding in entire northwestern part of Overijssel, causing 305 fatalities.

1830 Very severe winter

1838 Very severe winter

1845 Very severe winter

1845-1846 Peek years of European Potato Failure, harvest of potatoes in Northern Netherlands decreased by 57 percent, of rye by 47 percent, of wheat by 6 percent.

Supporting reference

1. Slicher van Bath BH. Mensch en land in de Middeleeuwen. Bijdrage tot een geschiedenis der nederzettingen in oostelijk Nederland. Assen: Van Gorcum; 1944.

* Data on winters are available from 1740 and are available from website Meteolink, https://www.meteolink.nl/weerhistorie-2/historische-winters/
